# It takes two to tango - how teacher-child interactions help advance children’s emotion knowledge

**DOI:** 10.3389/fpsyg.2025.1622163

**Published:** 2025-09-25

**Authors:** Oliver Hormann, Katharina Voltmer, Maria von Salisch

**Affiliations:** ^1^Technische Universität Braunschweig, Institute for Educational Science, Braunschweig, Germany; ^2^Leuphana University Lueneburg, Institute for Sustainability Psychology, Lueneburg, Germany

**Keywords:** early childhood education, teacher-led intervention, emotion knowledge, language support strategies, dialogue length, professional development, teacher-child interaction

## Abstract

Because young children’s emotion knowledge and language skills grow in tandem and contribute to their success in school, the Feeling Thinking Talking (FTT) teacher training, which addresses both areas, was developed. In this training, preschool and kindergarten teachers were taught to use language support strategies (LSSs) and responsive child-directed speech when talking about emotions with the children in their care. Whether these educational practices in teacher–child talk improve children’s emotion knowledge was examined with *N* = 275 children (Mage = 49.86 months, SD = 7.21, range = 35–66 months at t1) who were cared for by teachers in *N* = 16 training classrooms and *N* = 13 waitlist-control classrooms, which were nested in 13 centers. Children were individually tested on morphology, grammar, and emotion knowledge (EK) before (t1) and after the FTT training (t2). At t1 and t2, teacher–child interactions were videotaped and coded. Single-level models suggest that training group teachers used LSSs (input-oriented strategies and stimulation techniques) more often and involved children in longer dialogues than control group teachers at t2. Multilevel models show that dialogue length and feedback strategies at t1 and input-oriented strategies at t2 contributed to the explanation of gains in children’s EK over time (after controlling for covariates). Moreover, teachers’ use of input-oriented strategies at t2 that improved under the FTT training partially mediated the effect of the intervention on children’s EK growth. In addition, children’s (growing) language skills seem to mediate the effects of these educational practices on their EK. Ways in which educational practices affect emotion learning are discussed.

## 1 Introduction

Young children in Europe and North America spend many of their waking hours in centers of early childhood education in the company of their teachers and peers. Daily life in these centers provides numerous opportunities for them to feel and express emotions and to observe them in others (Alvarenga et al., 2020). Knowing how their peers and teachers look, sound, and behave when they are angry or disgusted, fearful or sad helps young children predict their behavior. Being aware of their own and others’ emotional states and being able to use words to communicate their feelings and needs to caretakers contributes to their adjustment (Houston et al., 2024). Emotion knowledge is an important ability for navigating the challenges of early childhood education.

### 1.1 Development of emotion knowledge

Emotion knowledge (EK) is also a key skill in all models of social–emotional learning (e.g., Denham and Brown, 2010). Between four and six years of age, children’s EK advances in rapid strides. Typically developing children learn how desires and (false) beliefs influence emotions and how display rules impinge upon the expression of emotion (Denham, 2023). According to a meta-analysis, children with an advanced understanding of emotions showed less internalizing and less externalizing problem behavior and were more socially competent (Trentacosta and Fine, 2010). Another meta-analysis corroborated that these children tended to like school better and were more successful in it, both socially and academically (Voltmer and von Salisch, 2017). Children’s knowledge of emotions thus seems to contribute to their social, emotional, and academic adjustment. This study focuses on how children acquire emotion knowledge.

Although some knowledge of emotions may be gained by observing others or by adults responding to emotions (e.g., Zinsser et al., 2023), the most prominent route seems to be through talking with adult family members (Zinsser et al., 2021) and with teachers in centers of early childhood education (e.g., Alvarenga et al., 2020; Zinsser et al., 2023). In the following, the focus will be on emotion talk in the context of teacher–child communication within these centers. The aim is to uncover whether young children’s emotion knowledge is advanced through specific interactions with their teachers in these centers.

### 1.2 Emotion talk advances children’s emotion knowledge

Emotion talk is a variant of mental state talk that has been studied in the context of parent–child interaction (e.g., Taumoepeau and Ruffman, 2008) and, more recently, with a focus on teachers’ interactions with the children in their care (e.g., Denham, 2023). Mental state talk directs children’s attention to emotions and helps them learn about states that are not readily observable (King and La Paro, 2015) and that may differ between individuals (Taumoepeau and Ruffman, 2008; Denham, 2023). Teachers’ emotion talk seems to advance children’s emotion knowledge. When teachers labeled the protagonists’ emotions during dialogical storybook reading, kindergartners tended to use more emotion words and knew more about the underlying thoughts (Bergman Deitcher et al., 2021). Observational studies have likewise confirmed that children’s understanding of emotions was predicted by teachers’ use of emotion words when reading picture books with them (Bassett et al., 2020). Sequential analyses showed that teachers’ questions about emotions during dialogical picture book reading stimulated children’s use of emotion words in their answers, which in turn was commented on by teachers, leading to dialogues on emotions, their causes, and consequences (Alvarenga et al., 2020). Emotion and mental state talk can thus advance children’s social and emotional learning via content that is embedded in contextual and sociocultural factors in their environments (Bell et al., 2024). Being engaged in more intensive talk about the mental states of others—be they real or fictional—was associated with a better understanding of emotions, according to a meta-analysis of parent–child conversation (e.g., Tompkins et al., 2018). Two recent reviews of early interventions with toddlers and preschoolers confirmed that emotion talk plays a central role in the social construction of emotion knowledge and comprehension, as well as prosocial behavior in childcare settings (Houston et al., 2024; Schapira and Grazzani, 2025). Through emotion talk, instruction, and modeling, children learn about emotion labels, problem-solving, and self-regulation (Denham, 2023; Zinsser et al., 2023). In summary, adults’ *use of language* in terms of how they talk with children about emotions has a direct effect on their knowledge of emotions. Understanding more about the effects of language, with a focus on the unique contributions of different features of children’s language environment to their development of EK, will help improve teachers’ professional training (Roberts et al., 2018). In the following, we will focus on the meaning of adults’ use of language support strategies (LSSs) and teacher–child dialogues for children’s advancement in EK.

### 1.3 Language acquisition and emotion knowledge with language support strategies

Adults’ use of LSSs facilitates children’s language acquisition (Nelson et al., 1996; Girolametto and Weitzman, 2002; Justice et al., 2008). LSSs fulfill three main functions: (1) to present frequent, highly informative linguistic *input*, (2) to *stimulate* children to produce output, and (3) to supply *feedback* on children’s utterances by selecting specific elements and presenting them in a corrected, expanded, or rephrased version (Hormann, 2024). *Input-oriented strategies* are made up of (partial) repetitions of the child’s or the adult’s own utterance and of parallel talk during which the adult verbalizes externally visible actions and parallel internal processes such as emotions or intentions (Gleitman et al., 1984; Raver et al., 2012). *Stimulation techniques* include different kinds of questions. Whereas open-ended questions elicit elaborate answers from children, closed questions (e.g., yes/no questions) not only provide them with low-threshold opportunities to engage in conversation but also with salient information about auxiliaries because these are presented in a noticeable position (Gleitman et al., 1984). Simple Wh-questions prompt the child to reproduce a familiar word. *Feedback strategies* are used when the adult provides a formally corrected, expanded, or reformulated version of the child’s non-adult-like or foreshortened utterance without explicitly calling it “ill-formed”. Whereas expansions extend children’s utterances by adding new syntactic or semantic information, reformulations pick up the preceding utterances of the child (or parts of them) and re-express them with morphological or syntactical changes in some parts of the sentence (Baker and Nelson, 1984; Girolametto and Weitzman, 2002; Taumoepeau, 2016). Supplementary Table A contains an overview of the LSSs that were used in this study.

Emotion talk in combination with LSSs seems to be an effective means to promote children’s EK. Input-oriented strategies may be beneficial because they can be expected to directly affect children’s understanding of emotion state language. Parallel talk, for example, attaches new vocabulary to actions, thoughts, or feelings the child cannot express yet. When learning the referents of emotion talk, children acquire the corresponding emotion concepts (Olson, 1988; Bartsch and Wellman, 1995; Ogren and Sandhofer, 2022). Indirect evidence for this position is found in studies indicating that children’s understanding of emotion-specific vocabulary contributes to their understanding of emotions (Streubel et al., 2020). This link is corroborated by studies that also found grammar to be highly correlated with emotion knowledge (e.g., Pons et al., 2003). Given the potential causal role of LSSs in children’s language acquisition, all three groups of LSSs could not only operate as *direct* catalysts of children’s acquisition of EK (in the case of input-oriented strategies, e.g., through the labeling of emotions that are otherwise not articulated) but also as an *indirect* factor that boosts children’s language competencies, which in turn enhance their EK. Knowing which type of LSS is effective in stimulating children’s EK can be used to improve programs of professional development for preschool and kindergarten teachers.

### 1.4 Language acquisition and emotion knowledge in extended dialogues

Children’s language acquisition is also advanced through participation in extended reciprocal (back-and-forth) communication (Dickinson and Smith, 1994; Romeo et al., 2018). Extended dialogues create opportunities for both conversation partners to align their attention and to collectively elaborate on their ideas and experiences. During these periods of sustained verbal exchanges, children’s and their partners’ utterances build on each other’s perspectives and cumulatively transcend their former understanding of the situation. This active co-construction implies a shift in language use from talking about things that happen in the “here and now” to dealing with the speakers’ perspective on events or topics. When expressing their thoughts and emotions about experiences (which include non-present events), adults and children access abstract words and advanced grammatical constructions (Bellagamba et al., 2022; Nelson, 2005). Nelson (2005, p. 40) claims that by attending to this sort of “representational language,” children not only become familiar with the rich linguistic toolkit but also gain insight into their own and their interlocutor’s consciousness, which includes their mental and emotional states. Another account for the impact of dialogues on EK development builds upon the idea that children’s understanding of emotions is a component of their broader social understanding (Tompkins et al., 2018). In line with the sociocultural approach of Vygotsky (1978) and the framework of dialogic thinking (Fernyhough, 2008), it can be assumed that children participating in extended dialogues come to internalize a plurality of perspectives on and representations of their own and others’ inner life, including emotional states, and thereby develop a deeper understanding of the “transactional” structure of emotions. That is to say, children learn that emotions do not have a meaning in themselves but have a meaning that depends on their particular viewpoint and that is related to and influenced by others’ perception of the situation.

In accord with these lines of reasoning, teacher–child dialogues can be assumed to stimulate children’s EK—directly and indirectly by improving their language skills. Empirical support for this conversational pathway comes from experimental (van Bergen et al., 2009; Ornaghi et al., 2015; Giménez-Dasí et al., 2017) and correlational studies (Dunn et al., 1991; Raikes and Thompson, 2008; van Bergen and Salmon, 2010). They demonstrate that the way adults talk about emotions (e.g., whether they engage children in discussions in which they elaborate on the causes of feelings) is just as important for gains in children’s EK as the simple use of verbal labels that refer to emotional states during the talk (Bell et al., 2024). Demonstrating the impact of longer back-and-forth exchanges extends evidence that was found for the beneficial nature of “sustained shared thinking” (SST) (Siraj-Blatchford et al., 2003) to the domain of emotions. Compared with extended dialogues, SST is a multidimensional concept that includes diverse behavioral factors, such as scaffolding, extending, discussing, modeling and playing. Some of these factors (e.g., extending) overlap with LSSs. The current study examines the paths through which teachers’ use of LSSs and longer dialogues may *directly* accelerate children’s growth in emotion knowledge. Since the data on teacher–child interaction and children’s EK cover two points in time, longitudinal as well as concurrent (parallel) associations with young children’s gains in EK will be analyzed.

Because LSSs and longer dialogues are both likely to foster children’s acquisition of emotion knowledge, they will be subsumed under the same label as “educational practices”. Yet, knowing the differential contribution of their potentially beneficial aspects to children’s gains in EK is decisive for improving teachers’ professional development. To understand the developmental value of different aspects of teachers’ educational behavior for optimizing teacher training, it is not sufficient to investigate whether they directly affect children’s progress. It is also necessary to study whether the training was able to induce a change in teachers’ behavior in accordance with the targets of the training. In other words, it is advisable to examine the role of educational (language-based) practices in mediating the effects of professional training on children’s EK development.

### 1.5 The FTT training

Strengthening teachers’ use of LSSs and enabling them to initiate sustained dialogues with children are core targets of the FTT training. Thus, data from the FTT training study can inform us about *how* these two distinct sets of educational practices may transmit the treatment’s effect on children’s development. In a previous study, both dimensions of educational practices have been examined mainly for their potential in transmitting the effects of the FTT training on children’s language acquisition (Hormann, 2024). For instance, both LSSs and the length of dialogues were shown to mediate the effects of teachers’ participation in the training on children’s understanding of syntax (as measured by the subtest “Sentence Understanding”) and on their linguistic productivity (as measured by the subtest “Morphological Rule Formation”). In another study, positive direct effects of the FTT training on children’s emotion knowledge have been established when language skills and other known covariates were controlled (Voltmer and von Salisch, 2022a). Yet so far, it has not been established whether the mediating role of educational practices in the transmission of the effects of the FTT training to young children’s language development extends to gains in their EK.

### 1.6 Mediating effects of children’s language abilities

A wholly absent feature of the studies cited above is the analysis of possible mediating effects of children’s language abilities. As discussed above, separate studies (including the first author’s own evaluation, Hormann, 2024) have shown that educational practices tend to accelerate children’s acquisition of verbal skills, and that these skills, in turn, are closely intertwined with their EK (e.g., Pons et al., 2003; Streubel et al., 2020). On the basis of this evidence, we hypothesize that the effects of teacher–child conversations on gains in children’s EK might be transmitted by children’s receptive and expressive language skills. Identifying the causal mechanism behind the relations between language-based educational practices and EK development is essential not only for our current understanding of *why* language-based educational practices work in supporting EK development, but also for our future understanding of *how* the training can be further adjusted to the learning requirements of the children. For instance, knowing whether the relation between teachers’ behavior and children’s EK growth is confounded by the latter’s language skills might be instructive in revising the training program in order to support children with limited (linguistic) capacities in the most effective way. For this reason, we examine whether (gains in) children’s understanding of sentences and (gains in the) application of morphological rules mediate the effects of educational practices on their gains in EK (and thus serve as second mediators) in the causal chain between teachers’ FTT participation and children’s EK development.

### 1.7 The present study

The novel contribution of the present study lies in examining the mediational pathways between the FTT training with teachers and children’s acquisition of emotion knowledge. This will be realized by examining (1) two distinct dimensions of teacher–child interactions (LSSs and dialogue length), which were core targets of the FTT training and served as main mediators within the overall transmission chain from the training to EK growth, and (2) two distinct language skills (sentence understanding and morphological rule formation) as mediators of the effects of educational practices on children’s gains in EK (with post-values being part of the second link within the overall transmission chain). The aim is to test the following hypotheses:

*H1:* The FTT training has a positive effect on gains in dialogue length and in the frequency of teachers’ LSS use (t2 scores, controlled for t1 scores).

*H2a:* Dialogue length and frequency of LSS use at t1 positively affect young children’s gains in emotion knowledge (t2 scores, controlled for t1 scores) over and above the influence of covariates (child age, gender, immigration background, and parents’ educational attainment).

*H2b:* Increases in dialogue length and frequency of LSS use (t2 scores, controlled for t1 scores) predict young children’s parallel gains in emotion knowledge over and above the influence of covariates (child age, gender, immigration background, and parents’ educational attainment).

*H2c:* The FTT training has an indirect effect on young children’s gains in EK (t2 scores, controlled for t1 scores) through its positive effect on changes in dialogue length and frequency of LSS use (t2 scores, controlled for t1 scores), which in turn affect children’s parallel gains in EK (t2 scores, controlled for t1 scores).

*H3:* Children’s gains in language abilities in terms of sentence understanding and morphological rule formation (t2 scores, controlled for t1 scores) mediate the effects of the state (t1 scores) and change of educational practices (t2 scores, controlled for t1 scores) on gains in children’s EK (t2 scores, controlled for t1 scores).

## 2 Materials and methods

### 2.1 Sample

*N* = 275 three- to five-year-old children (M_age_ = 49.86 months, SD = 7.21, range = 35–66 months at t1) were recruited from 13 centers of early childhood education. Children attended 29 classrooms located in and around towns in rural and urban areas in Northern Germany. A total of 46% of the children grew up in immigrant families, and 50% were female. In 37.4% of the families, at least one parent had a university degree; in 17.4%, one had a technical college degree; in 29.8%, a vocational qualification; and in 19.5%, less than 12 years of formal education. The allocation of the municipal, parochial, and company-run centers to the training group (TG) or the control group (CG) could not be realized through randomization due to the unwillingness of some centers to participate in the waitlist control group. To improve balance in possible unobserved confounders, we defined inclusion criteria for study participation (e.g., centers should have at least four groups and a share of 30–60% of children with German as their first language). This resulted in a sample of TG and CG centers that did not significantly differ in terms of stable (structural) features related to preschools’ educational quality (Kluczniok and Roßbach, 2014), such as the number of children in the classroom (M_TG_ = 25.25, M_CG_ = 21.31, *U* = 71.00, *p* = 0.145), teacher–child ratio (Median_TG_ = 10.47, Median_CG_ = 9.51, *U* = 79.00, *p* = 0.591), or teacher characteristics, for instance, their age in years (M_TG_ = 36.2, M_CG_ = 42.5, *p* = 0.091) (for more details see von Salisch et al., 2021). Based on multilevel regressions with children’s characteristics as outcomes and participation in the training as the binary predictor, differences between TG and CG in terms of children’s age (*p* = 0.841), gender (*p* = 0.115), immigration status (*p* = 0.739) and their parents’ educational attainment (*p* = 0.985) were non-significant.

### 2.2 Procedure

Data collection occurred with the positive ethical vote of one of the participating universities. The present study is based on data from the FTT training study (Voltmer et al., 2021; Voltmer and von Salisch, 2022a for details). Teachers in centers of early childhood education were trained to use LSSs while engaging the children in dialogues about internal states. The FTT training consisted of four modules (1–4) of about 8 h each and two modules (5 and 6) of about 4 h each, resulting in a total of 40 h. In modules 1 and 2, teachers were acquainted with basic knowledge about language acquisition and LSSs, with setting up teachable moments, and with creating extensive dialogues by using LSSs (e.g., open questions and expansions). In module 3, LSSs were applied to Emotion Talk, and in module 4, to Scientific Reasoning, which will not be discussed here. In accordance with the rules of dialogical reading, teachers were advised to follow children’s interests when naming and explaining emotions while reading picture books with emotional content and while reminiscing about emotional events. After module 3, teachers were asked to videotape specific interactions with the children. These videos were used to increase fidelity by fine-tuning teachers’ LSSs and Emotion Talk in one-to-one sessions with members of the research team. Module 5 aimed at the transfer of LSSs and Emotion Talk to other settings. The modules were scheduled over a period of about 6 months, with 1 month in between so that teachers could try out, practice, and integrate their new skills into their own style of teaching. Module 6 was a refresher module that was offered 3–6 months later.

**Figure 1:**
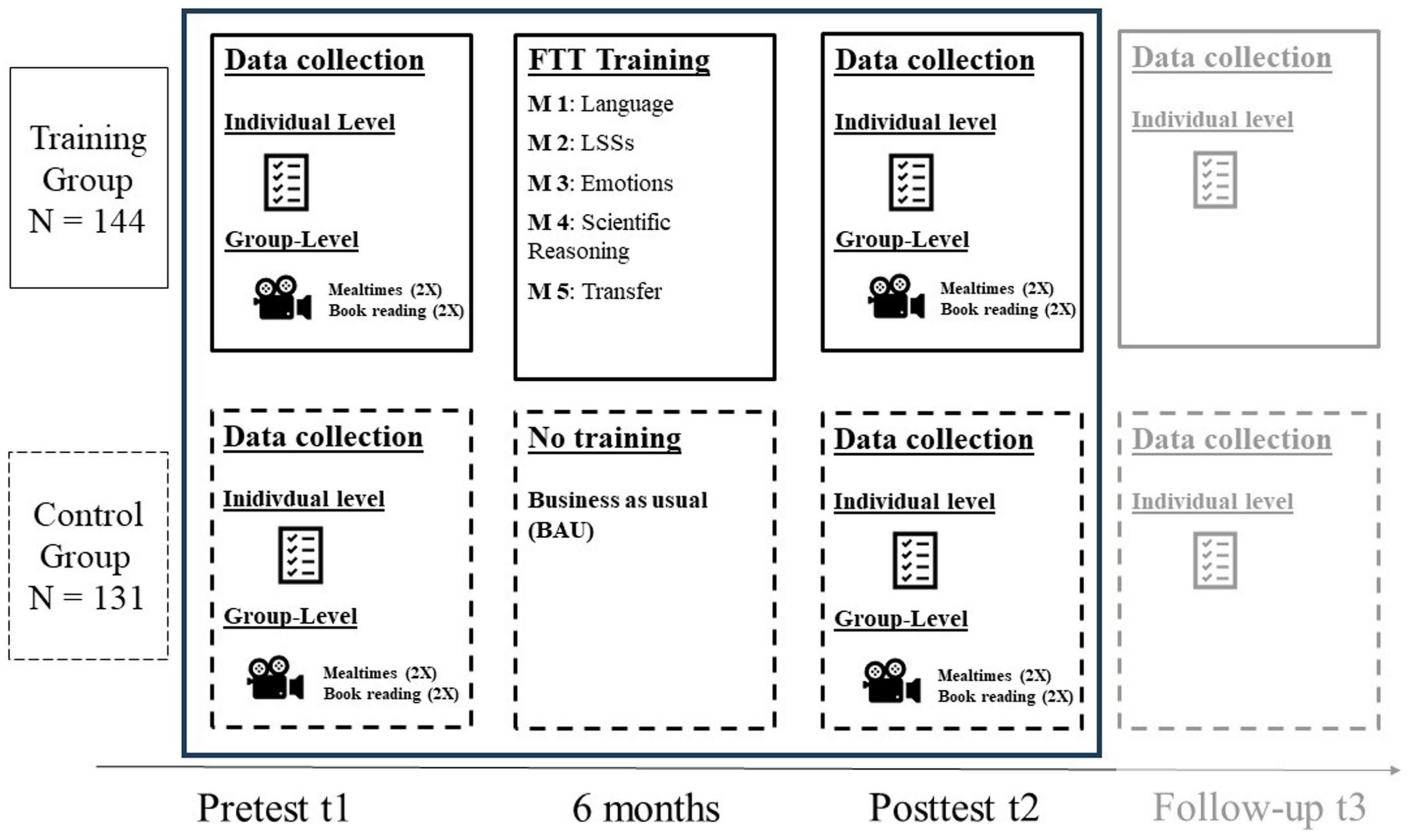
Design of the FTT evaluation study.

Participation was voluntary for the children and only took place when a child’s parent or legal guardian had given informed consent. The first measurement point (t1) took place before the beginning of the FTT training with the teachers. The second and third test sessions (t2 and t3) were conducted approximately 6 months and 1 year after t1, respectively. At all measurement points, children were tested individually in a quiet room at their kindergarten. Test administrators were trained undergraduate students. Data collection for each child at every measurement point included five blocks, each lasting about 30–40 min. Children completed a maximum of two blocks in 1 day, with breaks in between. Additionally, parents (at t1) completed a questionnaire about the family’s socioeconomic background. Because of technical difficulties, there were insufficient data on EK for t3, so only the t1 and t2 data were used in the current analyses. In each group, one of the usually two teachers who received the training was videotaped at both time points while interacting with the children in two settings (i.e., dialogical book reading and mealtimes). The other teacher, nonetheless, was invited to participate in the training (and usually did so). At t1 and t2, two videos per setting were recorded, resulting in four videos per time point and teacher (or classroom). The chosen settings include the planned setting of dialogic reading and the less structured setting of mealtimes. During the recordings, up to two members of the research team were present. Videos were usually recorded on two consecutive days with groups of two to six children. In order to keep contextual factors during dialogic reading constant, teachers’ choice of picture books was limited to two options. Figure 1 summarizes the research design.

### 2.3 Measures

#### 2.3.1 Children

**Figure 2:**
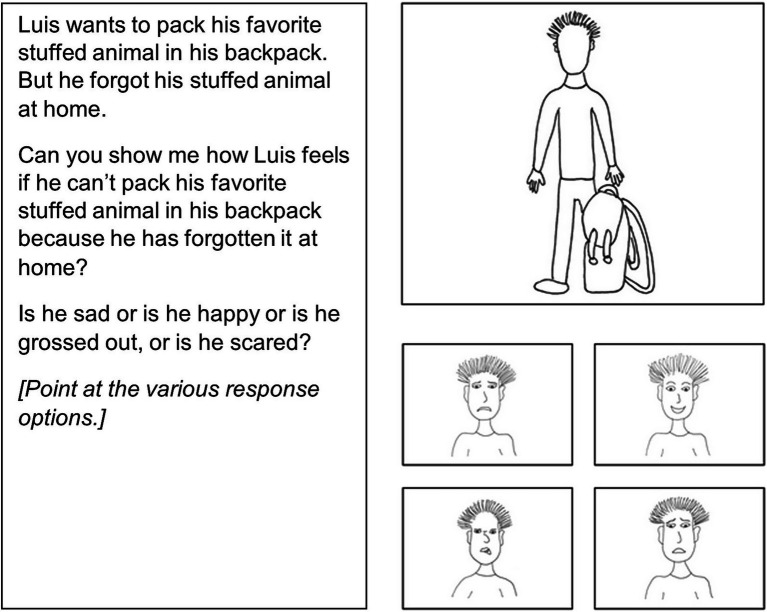
Sample item of ATEM 3-9.

*Emotion knowledge*. Emotion knowledge was assessed with a pre-final version of the “Adaptive Test of Emotion Knowledge for Three- to Nine-Year-Old Children” (ATEM 3-9; Voltmer and von Salisch, 2022b) at t1 and t2. It consisted of 50 items at t1 and 40 items at t2, which assessed the following components of emotion knowledge: (1) Recognizing emotions in the face (Recognition), (2) Identifying emotions in situations (Situations), (3) Identifying mixed emotions (Mixed Emotions), (4) Identifying emotions from desires (Desires), (5) Identifying emotions from (false) beliefs (Beliefs), and (6) Differentiating between shown and felt emotions (Felt/Shown Emotions). However, only items that were included in the final version of the ATEM 3-9 were analyzed in the present study, resulting in 32 items in total (with 5–6 items per component). Children’s total EK scores were created by summing up their answers to these items. The difficulty of the items increased, within and between components, as the story within which they are embedded progressed. A sample item is displayed in Figure 2 (more examples can be found in Voltmer and von Salisch, 2022b). Skipping and termination rules allowed less advanced children to work on fewer items. Children answered the items by pointing to one of four drawings of facial expressions: anger, sadness, fear, happiness, disgust, or surprise. The expected A posteriori (EAP) reliability coefficients—which can be interpreted similarly to Cronbach’s alpha (Adams et al., 2015)—ranged from 0.85 to 0.94 across the six components, based on a sample that included the t1 data from the present study (Voltmer, 2019). An overall EAP reliability coefficient was also calculated for the 32-item version of the scale used in this study, and this calculated reliability was high (EAP reliability = 0.93). Based on a smaller sample of t2 from the current study, we calculated an overall posttest EAP reliability of 0.87.

*Language competences:* Two subtests of the German *Language Development Test for 3- to 5-year-old children* (SETK 3–5; Grimm, 2015) were used to assess children’s levels of both receptive and productive language skills. Specifically, the selected tests are indicators of children’s ability to decode linguistic information embedded in sentences of varying grammatical complexity and their ability to apply the morphological rules of plural formation. The *Sentence Understanding (SU)* subtest covers mainly syntax but also calls for some lexical knowledge. Children were asked to carry out the experimenter’s instructions with various materials. Correct execution was awarded a point. Because there were 15 tasks of increasing grammatical complexity, a total of 15 raw points could be achieved; there was no stop criterion. Internal consistency of the SU subtest at t1 and t2 was high, with Cronbach’s alpha = 0.89 and 0.91. In the subtest on *Morphological Rule Formation (MR)* children were presented with 18 picture cards of common and uncommon, German nouns. The interviewer named the objects on the cards and elicited the plural form of it (“This is a [noun]. […pause…] Here are [number]…?”). The test was discontinued if children could not name the correct plural forms of the first 10 items. Two points were awarded for each correct plural form of a noun (one point for a partly correct form), resulting in a maximum of 36 raw points. Internal consistency of the MR subtest was high, with Cronbach’s alpha = 0.91 both at t1 and t2.

#### 2.3.2 Teacher–child interaction

*Language support strategies (LSSs)* used in four 5-min sequences of teacher–child interactions were randomly sampled from the 20 min of the raw videos recorded at t1 and t2. The resulting eight 5-min videos per teacher (four at each measurement time point) were fully transcribed and then coded for LSSs according to the subcategories outlined in Supplementary Table A. Videos instead of audio recordings were used mainly due to the necessity to assign all utterances correctly to the persons attending the situation. Because few feedback strategies were observed, expansions and indirect correctives were collapsed when calculating the interrater reliability. All videos were double-coded by pairs of student assistants who were uninformed about whether the videos depicted teachers of the TG or the CG. Cohen’s kappa was calculated on the basis of 125 min of the data corpus at t1. Except for open-ended questions, which had an insufficient level of reliability (kappa = 0.57) and were, therefore, excluded from further analyses, all other subgroups of strategies showed good to excellent kappa values: 0.75 (feedback strategies), 0.83 (repetitions), 0.85 (wh-questions), 0.92 (closed-ended questions), and 0.98 (parallel talk). Differences between coders were solved consensually with the help of the written manual. For each time point, the number of LSSs was first aggregated at the level of higher-order support functions (input, output, stimulation) and then averaged separately over the four 5-min sequences at t1 and t2. The resulting LSS scales thus reflect the mean quality of linguistic support that children in different classrooms experienced at the two time points. Averaging not only helps to map the linguistic quality of teacher–child interactions from two different contexts in a condensed way, but also minimizes the impact of random errors related to each of the four component measurements (Fleiss and Shrout, 1977).

*Dialogue Length* was operationalized as the mean number of turns within sequences of verbal interactions between teachers and children. A turn is defined as a shift in the role of the speaker that occurs whenever someone produces a verbal contribution to a conversation that is semantically contingent on the preceding verbal contribution of someone else and thereby extends the dialogue (Snow, 1996). A total of 82% of the videos were double-coded by pairs of student assistants who were unaware of the allocation of the videos to TG or CG. Interrater reliability was determined by an intraclass correlation coefficient based on the number of words within mutually identified dialogues (length of dialogue sequences) in 125 min of the video material. The overall coefficient was excellent (ICC = 0.96; t1 = 0.97; t2 = 0.95).

### 2.4 Control variables

*Child Age (t1).* Because language skills and EK grow with age, children’s age (in months) at baseline was controlled for.

*Child Gender.* Gender needs to be controlled because studies indicate that girls are often more advanced in their language abilities (e.g., Rinaldi et al., 2023) and sometimes in their EK (e.g., Denham, 2023).

*Child Immigration Background*. Because children with an immigration history are usually disadvantaged when it comes to acquiring German (as a second language) and EK in German (Hormann, 2024; Voltmer and von Salisch, 2019), children’s immigration status was controlled. A dichotomous variable was created indicating whether at least one parent was born in a country other than Germany based on parents’ information. If parents’ information was missing, teachers’ answers were used. In order to avoid multicollinearity—immigration background was strongly correlated with children’s language status (e.g., multilingualism, *r* = 0.71)—and to save degrees of freedom, we excluded indicators of child language and family economic status from the final list of covariates.

*Parents’ Highest Educational Attainment.* Information about parents’ educational background was obtained by asking them about their highest educational attainment. The scale comprised four ordered categories, ranging from “general education school leaving certificate after grade 10 or lower” (1) to “university degree or higher” (4). The indicator reflects the highest parental certificate within the family.

### 2.5 Statistical analyses

**Figure 3:**
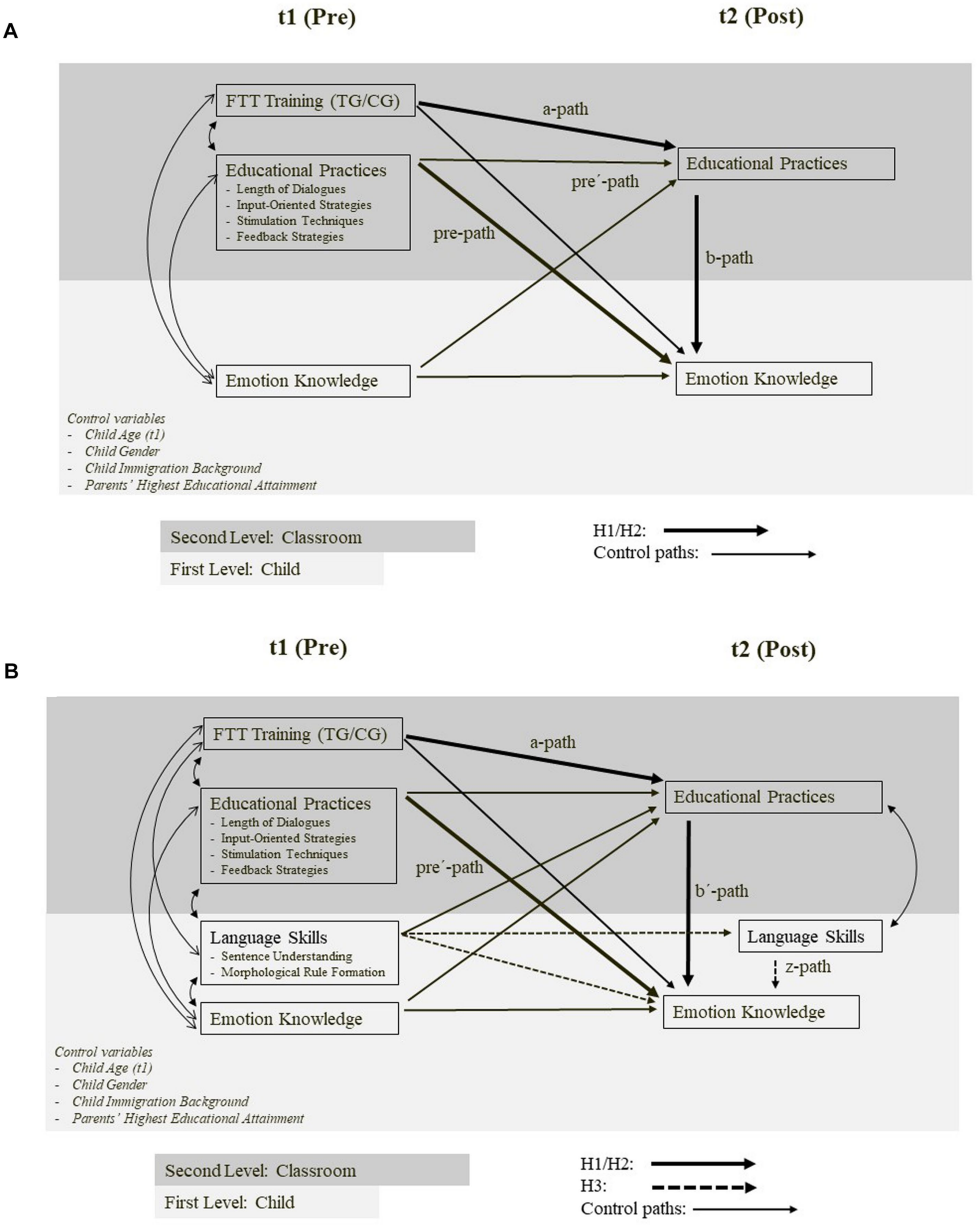
**(A)** Multilevel mediation model (H1 and H2). Control for Child Age (t1), Gender, Immigration background, and Parents’ Highest Educational Attainment. **(B)** Multilevel mediation model (H3). Control for Child Age (t1), Gender, Immigration background, and Parents’ Highest Educational Attainment.

For the outcome variable EK, about 18% of the data was missing at both t1 and t2 (the average percentage of missingness for *all* incomplete variables was 17%). Gaps in the data set were filled by means of multiple imputation (M = 50) with predictive mean matching (PMM) using chained equations and five potential donors (*k* = 5).^1^ Cluster membership at the classroom level was used as a fixed effect in the prediction. This has proven to be an effective means to avoid underestimation of intraclass correlations within multilevel data (Vink et al., 2015). Since multiple imputation relies on the assumption that the missing data mechanism is missing at random (MAR) or completely at random (MCAR), we performed Little’s missing completely at random test (MCAR test). Initially, we applied the test to all variables with missing data that were used in the imputation model, including incomplete variables of the analysis model. The *p*-value of the MCAR-test (*p* = 0.075) provides evidence that the pattern of missing values among the to-be-imputed variables is sufficiently independent of both observed and unobserved data (MCAR). Under missing at random conditions (MAR), which are created by the integration of auxiliary (complete) variables of the imputation model (including those of the analysis model), the p-value increases to 1.000. From this, it can be concluded that the missingness of the data depends only on the observed (external and accounted for) covariates (Li, 2013). Given the hierarchical structure of the data (children were nested in classrooms), our analytic models, as depicted in Figures 3A,B, take these different levels into account. Changes (or gains) in scores between t1 and t2 for both the independent and dependent variables were assessed by using the respective t1 scores as a covariate of the t2 scores, i.e., the ANCOVA approach (Valente and MacKinnon, 2017).

H1 asks whether participation in the FTT teacher training enhances teachers’ educational practices (by predicting the residualized scores of t2). Since both the training and the educational practices (LSSs and dialogue length) were located at the classroom level, the resulting a-paths of the mediation model (see Figure 3A) were estimated by single-level regressions with classrooms as cases based on a “between-effect model.” Between-effect models compute the relationship between the predictors and dependent variables using their group averages (or, in the case of binary predictors, the proportion of lower-level units that have the particular attribute). In this way, individual-level predictors (child age (t1), gender, EK (t1), immigration background, and parents’ education) were aggregated to the classroom level before being included as covariates in the prediction of the t2 values of teachers’ interactive behavior. The effects are reported in terms of partially standardized coefficients that represent the difference between TG and CG on a z-standardized dependent variable.

For H2 (both a and b), the scores of educational practices (LSSs and length of dialogues) from both t1 and t2 simultaneously predict EK at t2 with baseline outcome (EK at t1) as a covariate. Since H2 investigates the relation between upper-level predictors and a lower-level criterion, a two-level random-intercepts model was chosen to calculate the effects (Bliese, 2002). Coefficients of the t1 scores of LSSs and length of dialogues (pre-path, Figure 3A) represent the impact of rather stable group differences in educational practices (or *states*) prior to the training on *subsequent* changes in EK simply because the t1 values were not affected by the FTT training. The coefficients of the t2 scores (b-path, Figure 3A) accordingly represent the effect of the *changes* in educational practices from t1 to t2 on *parallel* changes in EK. Although the effect of educational practices’ t1 scores on EK’s t2 scores (residualized on EK’s t1 scores) fulfills the temporal requirements for causal conclusions more clearly, the parallel effects of the residualized t2 scores may map the causal order more accurately if, for example, the lag between cause and manifestation of the effect is very short (MacKinnon, 2008).

H2c predicts that children’s participation in the FTT teacher training enhances teachers’ educational practices, which in turn improve children’s EK, thereby creating an indirect link between the training and the gain in EK. As has been said, the coefficients of the educational practices’ residualized t2 scores represent the impact of changes in the quality of the educational practices on gains in EK (b-path, Figure 3A). As part of the previously discussed mediational chain, these t2 scores are themselves affected by the training (a-path). The significance of the mediation effects is tested with the “joint significance”-method and the “asymmetric distribution of products”-method, which have an excellent balance between the *α*-error rate and test power in the context of multilevel mediations (Pituch et al., 2005). The “joint significance” method is based on independent evidence for paths a and b. If both paths are significant, mediation via the indirect path (a → b) is considered “proven”. In contrast, the “asymmetric distribution of products” method generates a confidence interval (CI) for the product coefficient (a*b). If the confidence interval does not include zero, the indirect effect is significant (MacKinnon et al., 2002). The product coefficient is reported as a partially indirect effect that reflects the change in the dependent variable in terms of its standard deviations for a one-unit change in the predictor of interest, in this case, for the difference between being in the training versus control group (Miočević et al., 2018).

H3 examines the confounding influence of changes in children’s language abilities (residualized t2 scores) on the relation between t1 and t2 scores of educational practices and (residualized) t2 scores of EK. Since the relation between t2 scores of educational practices and EK growth is part of the overall mediation, any evidence of a confounding effect is, by implication, also evidence that the entire causal relation between the FTT training and EK growth via educational practices is partly due to children’s language gains. Therefore, the multilevel model for estimating the effects of t1 and t2 scores of educational practices on t2 EK (H2) was extended to include indicators of children’s language skills (SU and MR) from t1 and t2. The effects of educational practices net of the shared variance with children’s language skills were thus estimated (pre- and b-paths from H1 and 2 were replaced here by pre´- and b´-paths, see Figure 3B). With this decomposing strategy, borrowed from Ornaghi et al. (2015), at least a partial mediation (of the overall mediation) by children’s language skills can be inferred if it is established that (a) changes in children’s language skills add something to the prediction of changes in children’s EK (z-path, Figure 3B), and that (b) the direct effect of educational practices’ post scores (b´-path, Figure 3B) is substantially reduced after controlling for children’s language abilities compared to the b-path (Figure 3A). In order to evaluate the consequences of this (secondary) mediation effect on the overall mediation, the a-paths have to be adjusted for children’s language skills at t1 (in aggregated form).

Except for the “asymmetric distribution of products” method, which was performed with the R package “RMediation”, all statistical analyses were run in Stata 18. The separate testing of the a- and b-paths as part of the “joint significance” method, was carried out with the *xtreg, be* and the *mixed* command which are appropriate for fitting between- and multilevel models. The MACR-Test of Little is implemented in Stata with the *mcartest* command. The *estat vif* command was used to test for multicollinearity in the four upper-level models depicted in Table 1. The VIF statistics of the upper-level models are based on unimputed data, consisting of completely observed “true” level 2 measures of educational quality and mean-aggregated level 1 data of completely (child age, gender) and partly (EK, immigration Background, and Parents’ Highest Educational Attainment) observed level 1 indicators^2^. The VIF statistics for the multilevel model in Table 2 were calculated with the *mivif* command, which is applicable to imputed data and includes all predictors from Steps 2 and 3. The mean/highest VIFs for each of the models in Table 1 are 1.85/2.32, those for the “full” model in Table 2 are 2.03/3.39 and 2.17/3.39. Thus, none of the VIF statistics exceeds the established thresholds of multicollinearity, e.g., 4 or 10 (O’Brien, 2007).

**Table 1:** Prediction of changes in educational practices by the FTT training.

*Δ* Educational practices	Predictor: FTT training		
	Beta_ps_^#^ ≙ a-path	SE	*p*
Dialogue length	0.85	0.41	0.028
Input-oriented strategies	1.60	0.42	<0.001
Stimulation techniques	1.13	0.36	0.003
Feedback strategies	−0.13	0.45	0.386

*n* = 29 classrooms; control for TG/CG, scores of all dimensions of teacher-child interactions (t1), EK (t1), Child Age (t1), Gender, Immigration Background, and Parents’ Highest Educational Attainment; one-sided.

# Coefficients are partially standardized.

**Table 2:** Prediction of EK at t2 by educational practices (step 1) and child language skills (steps 2A and 2B).

Predictors	Step 1			Step 2A			Step 2B		
	Coefficients_fs_^#^	SE	Sign.	Coefficients_fs_^#^	SE	Sign.	Coefficients_fs_^#^	SE	Sign.
t1 Dialogue length	0.21	0.11	0.022	0.09	0.10	n.s.	0.14	0.10	n.s.
t2 Dialogue length	0.01	0.09	n.s.	−0.03	0.08	n.s.	−0.02	0.08	n.s.
t1 Input-oriented strategies	0.12	0.12	n.s.	0.16	0.11	n.s.	0.15	0.11	n.s.
t2 Input-oriented strategies	0.18	0.09	0.024	0.12	0.08	n.s.	0.14	0.08	0.042
t1 Stimulation techniques	−0.10	0.09	n.s.	−0.10	0.09	n.s.	−0.10	0.09	n.s.
t2 Stimulation techniques	0.12	0.11	n.s.	0.14	0.10	n.s.	0.15	0.10	n.s.
t1 Feedback strategies	0.14	0.08	0.040	0.09	0.07	n.s.	0.12	0.07	0.047
t2 Feedback strategies	−0.13	0.09	n.s.	−0.02	0.09	n.s.	−0.07	0.09	n.s.
t1 Sentence understanding	–	–	–	0.26	0.09	0.001	–	–	–
t2 Sentence understanding	–	–	–	0.19	0.09	0.017	–	–	–
t1 Morphol. rule formation	–	–	–	–	–	–	0.16	0.08	0.016
t2 Morphol. rule formation	–	–	–	–	–	–	0.14	0.07	0.034
R^2^ (based on LaHuis et al., 2014)	0.45			0.53			0.50		

*n* = 275 children; control for TG/CG, EK (t1), Child Age (t1), Gender, Immigration Background, and Parents’ Highest Educational Attainment; one-sided.

# Coefficients are fully standardized.

## 3 Results

In the following, the results of the data analyses are reported according to the research hypotheses H1 (Section 3.1), H2a, b and c (Section 3.2), and H3 (Section 3.3). Descriptive data and Pearson’s intercorrelations of the variables used in the analysis are presented in Supplementary Tables B, C.

### 3.1 Results for H1: the FTT training has a positive effect on gains in educational practices between t1 and t2

As can be seen in Table 1, participation in the FTT training exerted a partially standardized effect on changes in the length of teacher–child dialogues and in teachers’ use of input-oriented strategies and stimulation techniques between t1 and t2. Teachers who participated in the FTT professional development increased their use of input-oriented and stimulation strategies and engaged children in longer dialogues than their fellow teachers in the CG. The FTT training had no effect on the use of feedback strategies. Thus, H1 was confirmed for three of the four measures of educational practices.

### 3.2 Results for H2 (a–c): educational practices and the FTT training affect gains in children’s EK

#### 3.2.1 H2a: t1 educational practices affect the gains in children’s EK

Table 2 (step 1) displays the main results of hierarchical regression analyses about the impact of educational practices at t1 on EK at t2 while controlling for t1 EK as well as child and family covariates. T1 scores of dialogue length and feedback strategies contributed significantly to the change in children’s EK over time. H2a was thus confirmed for the LSS feedback strategies and the conversational feature of the length of dialogues. According to these results, children in classrooms of teachers who initially involved them in longer dialogues or who offered more feedback on their utterances made faster progress in their EK over time. The effect sizes of these two predictors, as measured by their (fully) standardized coefficients, were small to medium.

#### 3.2.2 H2b: t2 scores of educational practices affect the gains in children’s EK

Step 1 in Table 2 also shows that teachers’ increased use of input-oriented strategies at t2 predicted the simultaneous advancement of children’s EK with small to medium effect sizes. H2b was thus confirmed for input-oriented strategies. Children whose teachers expanded their verbalization of actions and inner states (including emotions) over time progressed more quickly in their EK.

#### 3.2.3 H2c: The FTT training has an indirect effect on children’s EK via changes in educational practices

According to the “joint significance” method, only educational practices that were (positively) affected by the FTT training (see Table 1) and whose t2 scores had an additional effect on gains in EK (Table 2, step 1) were considered to be mediators of the training. This condition was only met by the input-oriented strategies. Additionally, the results of the “asymmetric distribution of products” method showed a (partially standardized) indirect effect of 0.29 with a 95% CI ranging from 0.04 to 0.58. The fact that the CI did not contain the value zero indicates that the corresponding indirect effect (a*b) is significant (MacKinnon et al., 2002). The mediation effect indicates the influence of being in the TG (as opposed to the CG) on children’s z-standardized EK via changes in the educational practices the children were involved in. The CIs of all other indirect effects of educational practices covered zero (dialogue length: 0.01 [-0.12, 0.15], stimulation techniques: 0.13 [−0.07, 0.38], feedback strategies: 0.02 [−0.09, 0.15]). This implies that the corresponding indirect effects were non-significant. Thus, H2c was confirmed for input-oriented strategies (and not for the other three potential mediators).

### 3.3 Results for H3: influence of children’s language skills on their growing EK

The effects of educational practices that resulted after controlling for potential child-level language mediators (i.e., Sentence Understanding and Morphological Rule Formation) are presented in the middle and the right columns of Table 2. Adding t1 and t2 scores of sentence understanding (SU, step 2A) to the regression equation not only increased the amount of explained variance by eight percentage points but also caused a reduction of the coefficient of the input-oriented strategies t2 scores by about 30% which rendered the effect (including the overall mediational chain to which it belonged) insignificant. These results support the conclusion that the effects of changes in input-oriented strategies on gains in children’s EK (and the respective transmission of the training effect) came about in part through shared variance with SU. Adding SU also decreased the effect of the t1 scores of dialogue length and feedback strategies on gains in EK by 58 and 39%, respectively, so that the association with t2 EK became insignificant in either case.

When controlling solely for t1 SU (see Supplementary Table D), the effects of the gains in input-oriented strategies on gains in EK diminished by only 23% (*β* = 0.14, *p* = 0.049) and were still significant. Also, the overall mediational chain was, according to the “joint significance”-approach and after additional control for t1 SU in the a-paths (*β* = 1.61, *p* = 0.002, not otherwise depicted here), still intact (however, not according to the CI of the a*b-path [−0.01, 0.49], which included zero, not otherwise depicted here). After controlling for t2 SU only, the effect of t2 input-oriented strategies (in Table D) changed by the same degree (−24%), but the effect was non-significant due to the larger standard errors (*β* = 0.14, *p* = 0.064). Thus, t1 inter-group differences in children’s SU seemed to explain a certain portion of the t2 input-oriented strategies’ effect on EK (probably due to selection), but only the (additional) control for the t2 SU scores invalidated the effect. Also, the overall mediational chain became, according to both the “joint significance” method and the CI of the a*b-path ([−0.02, 0.47], not otherwise depicted here), non-significant. In addition, the effects of t1 dialogue length and feedback on children’s EK were reduced when controlling for t1 and t2 SU. Altogether, in the effects of educational practices on EK, a non-trivial interplay with children’s receptive grammar was observed.

In step 2B of Table 2, we entered children’s scores in Morphological Rule Formation (MR) as a (secondary) mediator (or confounder) of the effects of the educational practices. This increased the explained variance by about five percentage points compared to step 1. When controlling for t1 and t2 MR, the effects of t2 input-oriented strategies on EK declined by only 18% but remained significant (and showed no difference when changing the order of inclusion of t1 and t2 values). According to the “joint significance” method and because the CI of the corresponding indirect effect (0.23 [0.01, 0.50], not otherwise depicted here) did not contain zero, the mediation effect of input-oriented strategies was robust against controlling for children’s morphological skills. Additionally, t1 feedback strategies had a significant effect on EK in this model. Thus, in comparison with children’s receptive grammar skills, their morphological knowledge seems to exert a less important influence on the effects of input-oriented strategies on children’s growing EK. This is mirrored in the results of the t1 scores of dialogue length and feedback. Their effects on EK declined to a lesser extent and—in the case of feedback—remained significant when controlling for MR and not for SU (compare Table 2 steps 2A and B).

## 4 Discussion

The present intervention study underlines the potential of teacher-child interactions for promoting young children’s understanding of emotions. The indirect (mediational) effect via teachers’ input-oriented strategies suggests that the FTT training was successful in increasing teachers’ use of this subgroup of language support strategies (LSSs) (Hypothesis 1), which in turn contributed to children’s progress in their EK (Hypothesis 2b). This indirect effect was robust against alternative explanations for children’s advancing EK such as their age and gender, their immigration background, and their parents’ highest educational attainment, all of which had been statistically controlled for in the analyses. Establishing the (partial) mediation of the LSS “input-oriented strategies” on gains in children’s EK (Hypothesis 2c) supports theoretical proposals (e.g., Taumoepeau and Ruffman, 2008) and empirical results from a meta-analysis about the significance of parents’ mental state talk for children’s understanding of emotions (Tompkins et al., 2018), while extending these findings to elementary school teachers. On a more practical level, the mediation effect highlights the importance of validating children’s input when teaching young children about emotions (e.g., Zinsser et al., 2023) and corroborates the effect of the FTT training on children’s gains in EK (Voltmer and von Salisch, 2022a).

Trained teachers’ increased use of the input-oriented strategy “repetition of children’s and one’s own utterances” may have established a joint focus of conversation, validated children’s (verbal) expressions of emotions, and encouraged them to talk about their own feelings and those of other children. For some children, emotions may have become an “allowed” topic of conversation with their teachers. Teachers’ more frequent use of the input-oriented strategy “parallel talk about inner states” in their child-directed speech may have elaborated children’s emotion concepts by providing words for facial expressions, physical responses (such as blushing), and typical situational antecedents and appraisals of emotions. Both kinds of input-oriented strategies aggregate disparate emotion information into meaningful linguistic categories. Having concepts and words for emotions makes it easier for children to recognize them when they occur (Ogren et al., 2024; Hoemann et al., 2019). Teachers’ use of input-oriented strategies may have stimulated young children’s emotion knowledge, which at ages three and four mainly revolves around naming facial expressions of emotions (Voltmer and von Salisch, 2022a) and learning about contingencies between situational antecedents and ensuing emotions (Alvarenga et al., 2020).

Teachers’ use of the linguistically more advanced LSS feedback strategies was observed more rarely overall than input-oriented strategies. Nevertheless, the more often teachers used feedback strategies before the training (t1), the more the children in their groups progressed in their emotion knowledge over time (when controls were in place). One explanation for this predicted result in Hypothesis 2a is that the feedback strategy *expansion* (which repeats children’s utterances and adds new, possibly explanatory, elements to them) can be used to enlarge their EK, for example, by combining situational antecedents and facial expressions of emotions into meaningful categories (Ogren and Sandhofer, 2022). These categories are the building blocks of EK. Teachers’ frequent use of these LSSs may thus have contributed to the gains in children’s EK.

Another interindividual difference between teachers that affected children’s development of EK was the length of their dialogues at t1 (Hypothesis 2a). That sustained exchanges of ideas between teachers and children tend to stimulate conceptual development is well known from research on sustained shared thinking (e.g., Siraj-Blatchford et al., 2003) and on dialogical book reading (e.g., Bassett et al., 2020; Bergman Deitcher et al., 2021). Children who initially attended classrooms of teachers who managed to engage them in extended conversations made more progress in their EK than their agemates whose teachers had shorter interchanges with them. In the area of emotions, dialogues can be used to co-construct more advanced emotion-related concepts, such as the meaning of mixed emotions, more complex situational antecedents, or differences between subjectively experienced and outwardly expressed emotions (display rules). In fact, post-hoc follow-up tests over the components of the ATEM 3-9 revealed that mixed emotions and situational antecedents were the components of EK in which the TG children of this sample (in the FTT data set) made more progress over time than their agemates in the CG (Voltmer and von Salisch, 2022a).

Teachers’ dialogues marginally increased in length during the FTT training, but these gains did not contribute to the explanation of the gains in children’s EK over time beyond the impact of prior differences in the length of dialogues. It seems most likely that the missing effects are due to the fact that the *changes* in dialogue length between t1 and t2 had a less persistent impact on children’s development than its baseline level (t1), which represents prior *interindividual differences* in the connectedness of teacher–child interchanges (i.e., without interference from the treatment). The causal mechanism through which dialogues affect children’s EK development may run like this: Participating in dialogues stimulates children to construe an internal representation of the external structure provided by the conversation (Fernyhough, 2008). In a Vygotskian view (Vygotsky, 1978), this learning process supposedly spans over longer periods of time (and multiple experiences of dialogically enriched interactions with teachers) in comparison with the learning process triggered by linguistic input that enables a direct mapping of lexical items onto the corresponding emotion concepts (Bartsch and Wellman, 1995; Ogren and Sandhofer, 2022). In summary, improving teachers’ ability to verbally pick up on children’s emotions (or model their own emotions) seems to ‘pay off’ immediately, given the effects of short-term changes in teachers’ use of input-oriented LSS between t1 and t2 on the contemporaneous gains in EK. In contrast, improving teachers’ abilities to engage children in extended dialogues may represent an ‘investment’ in children’s EK that ‘pays off’ only after these abilities have unfolded their beneficial effects over time.

The present study underlines the importance of children’s language abilities to their EK development, as predicted in Hypothesis 3. Controlling for two measures of children’s receptive and productive skills (Sentence Understanding and Morphological Rule Formation) reduced the direct impact of educational practices on children’s progress in EK and weakened the overall mediational chain. This result speaks for the close relations between children’s language abilities and their EK. It corroborates the idea that supporting children’s language development may also foster their EK development in the long run. Leaving this general remark aside, we found evidence that children’s (gains in) linguistic understanding tended to play a larger role in the mediation than their expressive grammar, probably because these receptive skills relate more directly to children’s abilities to participate in verbal exchanges about inner processes and, consequently, to their development of the corresponding cognitive representations. In addition, children’s ability to apply the rules of plural formation may not be a precise enough measure of their productive grammar skills.

Because of the intertwined development of language and emotion knowledge (Voltmer and von Salisch, 2025), the FTT training addresses both domains of learning with an integrated approach. This teacher training has been shown to promote both children’s language skills in different areas (e.g., SU and MR, see Voltmer et al., 2021) and their EK (Voltmer and von Salisch, 2022a). The FTT training aims at the professional development of early childhood teachers because, on the one hand, they are the catalysts for young children’s development of EK (Curby et al., 2022). On the other hand, they tend to differ in the quantity and quality of their emotion talk (King and La Paro, 2015; Alvarenga et al., 2020; Zinsser et al., 2023) and their mental state language in general (Andrews et al., 2020). Teachers are a suitable target group for interventions because they can make use of the many opportunities for (emotion) talk with children that arise during the day. In these dialogues, they can tailor their verbal input (on emotions) to children’s individual proficiency in language and EK in a way that stimulates their development.

Concerning the mediating role of children’s linguistic abilities in the causal chain between teachers’ use of input-oriented strategies and children’s EK development, those with a low command of SU or MR may profit most from extended conversations and from the provision of linguistic input that connects emotion words with actions or situations. The reason for this assumption is that input-oriented strategies such as parallel talk are predestined for use vis à vis children with less mature linguistic abilities (e.g., input-oriented strategies provide linguistic information within the child’s attentional focus about what the child is interested in but not able to express herself). Because emotion talk is part of teachers’ interactions with individual children or groups of them during the day, nearby children who are not addressed (but overhear these conversations) may profit from teachers’ explanations as well. Teaching teachers how to engage children with limited linguistic abilities in extended dialogues by using input-oriented strategies that offer emotionally meaningful content should be part of every teacher training.

Three-to-six-year-olds are an excellent target group for language-based interventions because they can take advantage of the neural plasticity in their brains. This may result in reducing the SES-informed differences in brain structures that have accumulated over their first years of life (Merz et al., 2019). Language-based interventions will thus contribute to school readiness for less advantaged children. Because learning in the early years is a very social endeavor (Denham and Brown, 2010), improvements in their language skills (von Stumm et al., 2020) and in their EK are both likely to contribute to their academic success (Voltmer and von Salisch, 2017).

### 4.1 Strengths

Strengths of the current study include the fact that the effects of the FTT training on teachers’ interactive behavior could be demonstrated by their increased use of LSSs, as well as by their engagement in extended dialogues with the children. Multilevel mediation analyses that analyzed the impact of educational practices on individual outcomes (under control of the direct effect of the treatment) suggest that teachers’ baseline differences in using feedback strategies and in initiating sustained dialogues with children, as well as their increased use of input-oriented strategies (post-training) were responsible for children’s gains in EK over time. These results further suggest that teachers’ use of input-oriented strategies is a potential transmission belt for interventions targeting children’s learning about emotions. Strong points of the study include that it is based on observations of teacher-child interactions in both planned and unplanned situations (beyond shared book reading), that it combined these observations with objective tests on EK and language skills, and that it excluded alternative explanations for the increase in children’s EK by controlling for third variables. Noteworthy is also the sample with a large proportion of children with foreign-born parents (45%), which corresponds to their proportion in representative samples from this region of Germany (e.g., Bock-Famulla et al., 2017).

### 4.2 Limitations

Limitations include the lack of randomization of the centers for early childhood education. Although the centers of the TG were, as already mentioned, comparable to those of the CG in many respects (e.g., teacher-child ratio, teacher qualifications, and children’s family backgrounds), it cannot be ruled out that TG centers may have differed in other yet unmeasured third variables. A follow-up measurement was missing. Teachers’ interactive behavior was measured by taking “snapshots” of their performance, which do not allow us to relate their behavior to children’s individual development. That is also why cross-level interaction effects tend to camouflage the contextual variance and the temporal instability of teachers’ performance. It cannot be excluded that some children may have received a higher quantity (or quality) of language input from their teachers than others. A drawback was also that the study’s primary focus was on teachers’ general verbal interactions with the children and not specifically on their emotion talk, which seems to occur more rarely (Zinsser et al., 2023). The focus on dialogue length may also have obscured differences in the quality of these conversations. Therefore, a more fine-grained coding scheme is needed in future studies. Additional moderators of teacher-child interactions, such as teachers’ depressive symptoms (Granger et al., 2023), should be considered as well.

### 4.3 Conclusion

The present study underlines the importance of the process quality of teacher-child interactions for the transmission of EK to the next generation. A future study with a larger sample of children would allow for more detailed analyses of the paths between the quantity and quality of teacher-child interactions and children’s growing EK, e.g., regarding the moderating effects of children’s language proficiency or their families’ socioeconomic status and migration history. Further theoretical and empirical analyses are needed to uncover whether children’s language abilities tend to enhance their emotion knowledge, whether emotion knowledge stimulates their language development, or whether both develop in tandem. In general, future studies are needed to corroborate the efficacy of the FTT training on children’s EK for children of different ages and over longer periods of time.

## Data Availability

The raw data supporting the conclusions of this article will be made available by the authors without undue reservation.

## Footnotes

^1^The imputation model included all variables related to language or EK, the kindergarten site, as well as all variables that were included in the model used to evaluate the hypotheses.

^2^We were not able to use imputed level 1 data for EK, immigration Background, and Parents’ Highest Educational Attainment because stata does not offer a routine to aggregate this information in all 50 data sets and calculate a common VIF value. Since the reported amount of multicollinearity is small and the majority of the covariates used were fully observed, we do not expect any serious distortion of the results.
