# Supplementary material for: It takes two to tango - how teacher-child interactions help advance children’s emotion knowledge
Source: Front Psychol. 2025 Sep 25;16:1622163. doi: 10.3389/fpsyg.2025.1622163 (PMC12533283; doi:10.3389/fpsyg.2025.1622163)
Supplement: Supplementary file 1 [file Table_1.docx]

**Table A** (Non-exhaustive) overview of language support strategies

| **Input-oriented strategies** | **Stimulation techniques** | **Feedback strategies** |
| --- | --- | --- |
| **Parallel talk**  Description of the child’s or one’s own action  (Teacher: “You are smiling”)  Description of the child’s or one’s own inner state  (Teacher: “I am sad that all the cake is eaten”)  **Repetition**  (Partial) Replication of the child`s utterance  (Child: “I like chocolate.”, Teacher: “Yes, chocolate.”)  Partial Replication of own utterance (Teacher: “A fish. A flying fish.”) | **Open-ended questions**  Non-specific request (Teacher: “What do you feel?”)  **Closed-ended questions**  Can be answered with “yes” or “no” (Teacher: “Are you sad?”) or (partial) repetition of a phrase (Teacher: “would you like to play in the playground or in the activity room?”)  **Simple Wh-questions**  Can be answered with the name or label of an object  (Teacher: “What is that?”) | **Expansions and re-formulation**  (Partial) Repetition of child’s preceding utterance with added and/or syntactically changed elements (Child: "I draw." – Teacher: “You draw a picture, didn’t you?").  Re-expression of child’s utterances with morphological or syntactical changes (Child: “I want to eat my lunch. I`m hungry” – Teacher: “Because you are hungry you want to eat your lunch”)  **Indirect corrective feedback**  Rectification without explicit identification of the error (Child: “He see me.”, Teacher: “He sees you.”) |
