# Supplementary material for: It takes two to tango - how teacher-child interactions help advance children’s emotion knowledge
Source: Front Psychol. 2025 Sep 25;16:1622163. doi: 10.3389/fpsyg.2025.1622163 (PMC12533283; doi:10.3389/fpsyg.2025.1622163)
Supplement: Supplementary file 2 [file Table_2.docx]

**Table B** Descriptive statistics and correlations for level 1 study variables

| Variables | n | M | SD | (1) | (2) | (3) | (4) | (5) | (6) | (7) | (8) | (9) | (10) |
| --- | --- | --- | --- | --- | --- | --- | --- | --- | --- | --- | --- | --- | --- |
| (1) t1 Emotion Knowledge | 275 | 7.06 | 5.07 |  |  |  |  |  |  |  |  |  |  |
| (2) t1 Sentence Understanding | 275 | 6.51 | 4.55 | .52^***^ |  |  |  |  |  |  |  |  |  |
| (3) t1 Morphol. Rule Formation | 275 | 14.32 | 10.03 | .50^***^ | .68^***^ |  |  |  |  |  |  |  |  |
| (4) t2 Emotion Knowledge | 275 | 10.33 | 6.63 | .57^***^ | .63^***^ | .54^***^ |  |  |  |  |  |  |  |
| (5) t2 Sentence Understanding | 275 | 8.12 | 4.66 | .47^***^ | .78^***^ | .69^***^ | .59^***^ |  |  |  |  |  |  |
| (6) t2 Morphol. Rule Formation | 275 | 17.73 | 10.23 | 47^***^ | .66^***^ | .70^***^ | .51^***^ | .72^***^ |  |  |  |  |  |
| (7) Child Gender | 275 | 0.49 | 0.50 | -.03 | -.07 | -.07 | -.05 | .00 | -.05 |  |  |  |  |
| (8) Child Age (t1) | 275 | 49.86 | 7.21 | .32^***^ | .22^***^ | .12 | .32^**^ | .16^*^ | .08 | -.14^*^ |  |  |  |
| (9) Child Migration Background | 275 | 0.45 | 0.50 | -.23^***^ | -.41^***^ | -.35^***^ | -.26^***^ | -.48^***^ | -.36^***^ | -.07 | .09 |  |  |
| (10) Parents’ Highest Educational Attainment | 275 | 2.77 | 1.11 | .14 | .41^***^ | .36^***^ | .19^**^ | .38^***^ | .35^***^ | -.01 | -.25^**^ | -.33^***^ |  |
| (11) TG vs. CG | 275 | 0.52 | 0.50 | -.08 | -.03 | -.04 | .02 | .04 | .04 | .10 | -.01 | -.05 | -.02 |
| *Note. ^***^ p< .001, ^**^ p< .01, ^*^ p< .05, two-sided* | | | | | | | | | | | | | |
