# Supplementary material for: It takes two to tango - how teacher-child interactions help advance children’s emotion knowledge
Source: Front Psychol. 2025 Sep 25;16:1622163. doi: 10.3389/fpsyg.2025.1622163 (PMC12533283; doi:10.3389/fpsyg.2025.1622163)
Supplement: Supplementary file 3 [file Table_3.docx]

**Table C** Descriptive statistics and correlations for level 2 study variables

| Variables | n | M | SD | (1) | (2) | (3) | (4) | (5) | (6) | (7) |
| --- | --- | --- | --- | --- | --- | --- | --- | --- | --- | --- |
| (1) t1 Dialogue Length | 29 | 3.38 | 0.94 |  |  |  |  |  |  |  |
| (2) t1 Input-Oriented Strategies | 29 | 5.10 | 1.74 | .50^**^ |  |  |  |  |  |  |
| (3) t1 Stimulation Techniques | 29 | 12.97 | 2.71 | .10 | .20 |  |  |  |  |  |
| (4) t1 Feedback Strategies | 29 | 2.81 | 1.40 | .22 | .17 | .26 |  |  |  |  |
| (5) t2 Dialogue Length | 29 | 5.08 | 2.00 | .17 | -.08 | -.03 | .31 |  |  |  |
| (6) t2 Input-Oriented Strategies | 29 | 5.85 | 2.09 | -.19 | -.10 | -.21 | .02 | .34 |  |  |
| (7) t2 Stimulation Techniques | 29 | 15.30 | 3.96 | -.25 | -.34 | .39* | .23 | .16 | .30 |  |
| (8) t2 Feedback Strategies | 29 | 3.32 | 1.56 | -.32 | -.54^**^ | .05 | .10 | .17 | .10 | .16 |
| *Note*. *^***^ p< .001, ^**^ p< .01, ^*^ p< .05, two-sided* | | | | | | | | | | |
