# Supplementary material for: It takes two to tango - how teacher-child interactions help advance children’s emotion knowledge
Source: Front Psychol. 2025 Sep 25;16:1622163. doi: 10.3389/fpsyg.2025.1622163 (PMC12533283; doi:10.3389/fpsyg.2025.1622163)
Supplement: Supplementary file 4 [file Table_4.docx]

**Table D** Supplementary results to Table 2 (steps 2A), separately controlled for t1 and t2 Sentence Understanding

|  | Step 2A (only SU t1) | | | Step 2B (only SU t2) | | |  |
| --- | --- | --- | --- | --- | --- | --- | --- |
|  | Beta_fs_ | SE | Sign. | Beta_fs_ | SE | Sign. |  |
| t1 Dialogue Length | 0.13 | 0.10 | n.s. | 0.10 | 0.10 | n.s. |  |
| t2 Dialogue Length | -0.02 | 0.08 | n.s. | -0.02 | 0.09 | n.s. |  |
| t1 Input-Oriented Strategies | 0.16 | 0.11 | n.s. | 0.14 | 0.12 | n.s. |  |
| t2 Input-Oriented Strategies | 0.14 | 0.08 | .049 | 0.14 | 0.09 | n.s. |  |
| t1 Stimulation Techniques | -0.10 | 0.09 | n.s. | -0.09 | 0.09 | n.s. |  |
| t2 Stimulation Techniques | 0.14 | 0.10 | n.s. | 0.12 | 0.11 | n.s. |  |
| t1 Feedback Strategies | 0.10 | 0.07 | n.s. | 0.10 | 0.08 | n.s. |  |
| t2 Feedback Strategies | -0.04 | 0.09 | n.s. | -0.06 | 0.09 | n.s. |  |
| t1 Sentence Understanding | 0.36 | 0.07 | <.001 | - | - | - |  |
| t2 Sentence Understanding | - | - | - | 0.33 | 0.07 | <.001 |  |
| R² _(based on LaHuis et al., 2014)_ | .52 |  |  | .50 |  |  |  |
| *Note: n= 275 children; control for TG/CG, EK (t1), Child Age (t1), Gender, Immigration Background, and Parents’ Highest Educational Attainment; one-sided*  *^#^ Coefficients are fully standardized* | | | | | | | |
